# Combined Antegrade-Retrograde Dilation of Radiation-Induced Benign Esophageal Stenosis

**DOI:** 10.14309/crj.0000000000000947

**Published:** 2023-01-25

**Authors:** Wasef Sayeh, Albert Tsang, Jordan Burlen, Sami Ghazaleh, Azizullah Beran, Ziad Abuhelwa, Tarik Alhmoud, Muhannad Heif

**Affiliations:** 1 Department of Internal Medicine, University of Toledo, Toledo, OH; 2 Department of General Surgery, Toledo Hospital, Toledo, OH; 3 Department of Gastroenterology, University of Toledo, Toledo, OH; 4 Depratment of Gastroenterology, Toledo Hospital, Toledo, OH

**Keywords:** CARD, septotomy, esophageal stenosis, needle knife

## INTRODUCTION

Head and neck cancer is a common malignancy with almost 450,000 newly diagnosed cases worldwide each year.^1^ Radiation therapy plays a major role in the treatment of oropharyngeal cancers, which can be used alone or in combination with surgery and chemotherapy.^2^ Approximately 0.8%–2.6% of patients develop esophageal stenosis and strictures, but the incidence of these adverse events can increase up to 37% in patients who received chemotherapy and radiation therapy.^3^ Partial esophageal stenosis or strictures, where there is incomplete closure of the lumen, are usually treated with antegrade dilation techniques using Maloney bougie dilators, balloon dilators, or Savary dilators.^4^ The treatment of complete strictures, where there is a complete occlusion of the lumen without a pinhole, is not as straightforward. Some case series described the use of a combined antegrade and retrograde dilation (CARD) technique to treat complete esophageal stenosis.^5^

In this report, we describe a novel treatment method of radiation-induced complete esophageal stenosis that was treated with combined antegrade-retrograde dilation with the use of needle knife for septotomy.

## CASE REPORTS

This is a case of a 76-year-old man who was recently diagnosed with oropharyngeal squamous cell carcinoma at the base of the tongue, with metastatic disease to a submandibular lymph node-stage 3 (cT4, cN3, cM0, and p16+). He was treated with concurrent weekly chemoradiation therapy. He was started on tube feeds through percutaneous endoscopic gastrostomy (PEG) tube at the time of diagnosis because of decreased oral intake and fears of aspiration. The patient continued using the PEG tube throughout the treatment duration for almost 2 months. Three weeks after the last radiation session, he presented with symptoms suggestive of esophageal dysphasia. He was evaluated by a video swallow study that showed aspiration with thin consistency and regurgitation in the proximal esophagus with findings suggestive of luminal narrowing because of soft tissue edematous changes. The patient was therefore scheduled for an endoscopic intervention.

**Figure 1.**
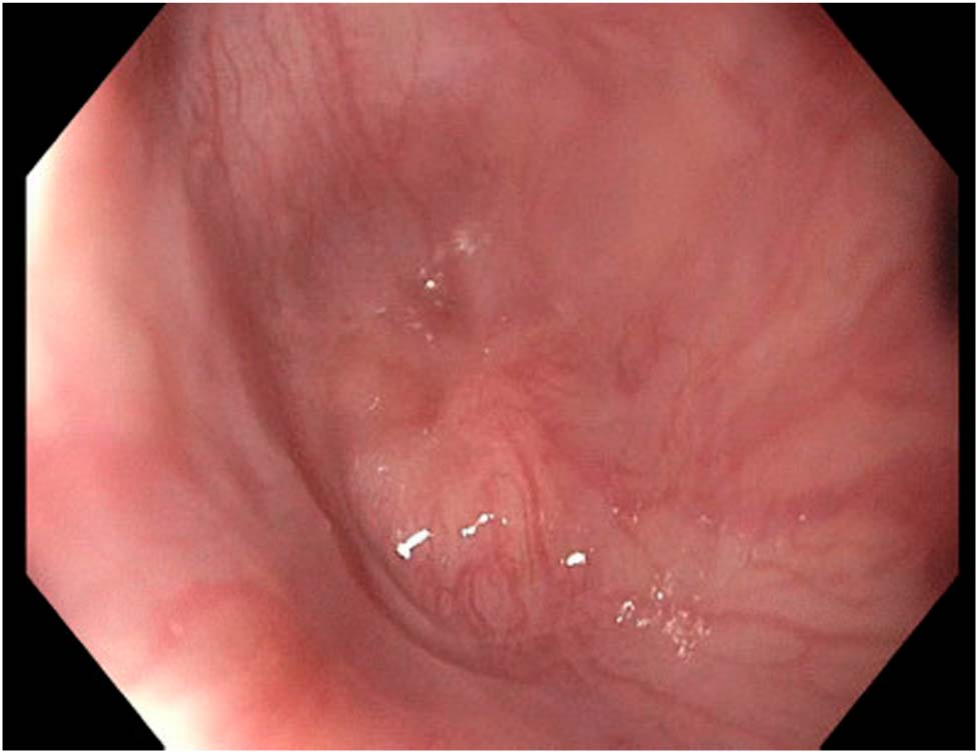
The esophageal septum/stenosis.

**Figure 2.**
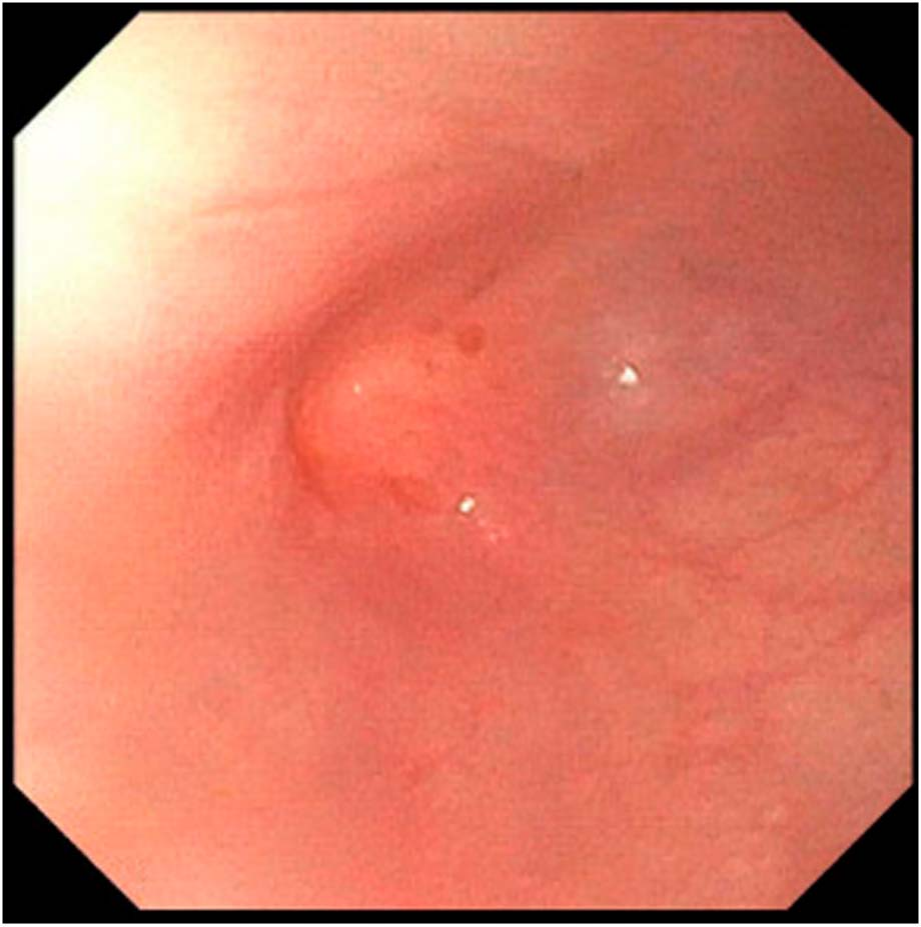
Transillumination seen by the ultraslim scope from below.

**Figure 3.**
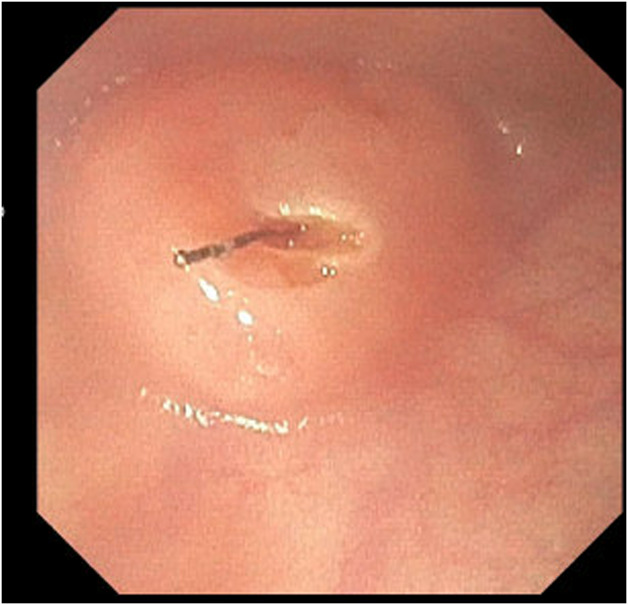
Needle knife septotomy.

**Figure 4.**
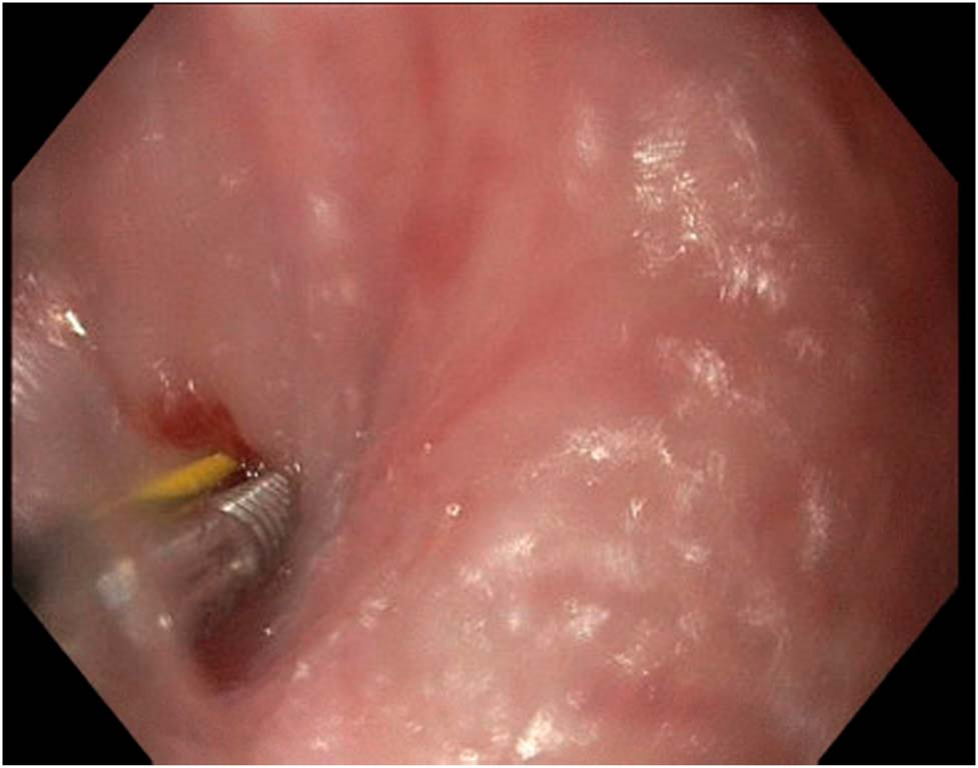
Savary guidewire advancement through the septotomy under the direct vision of the adult scope.

**Figure 5.**
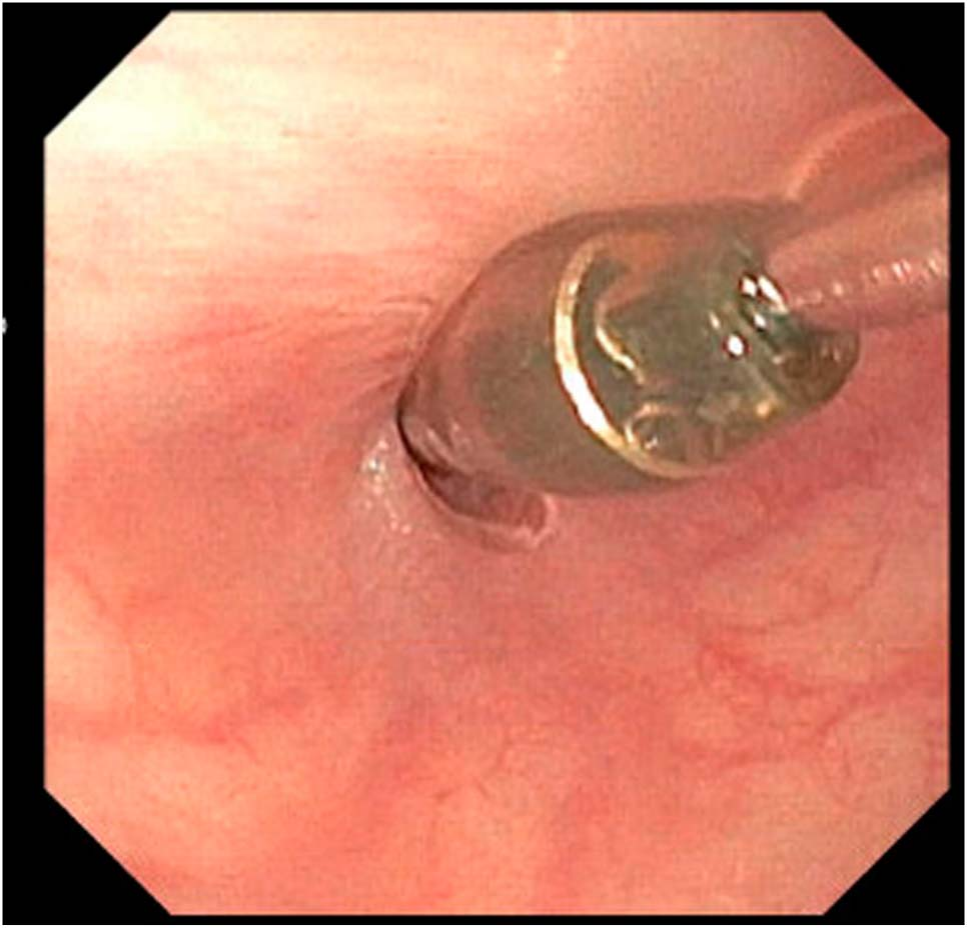
Esophageal dilation using the savary dilator.

**Figure 6.**
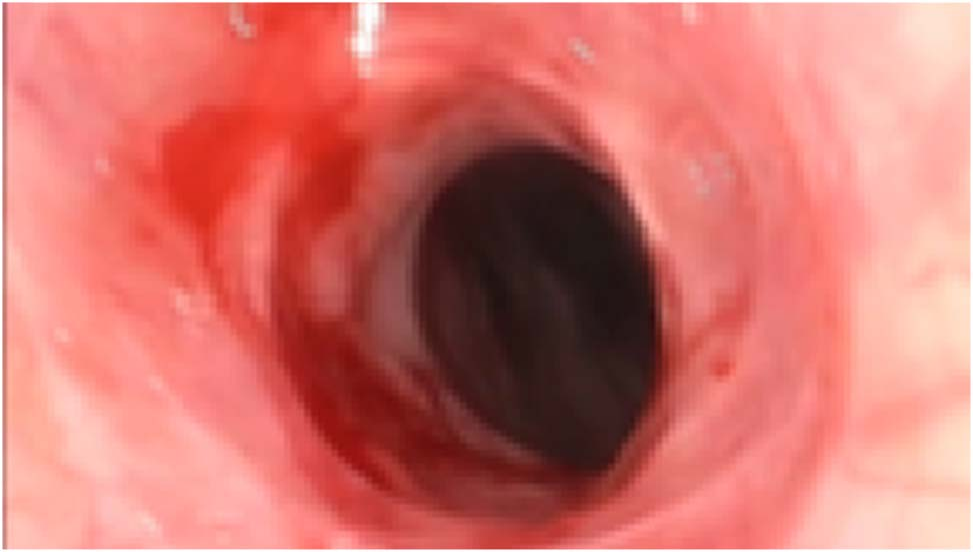
Repeat EGD showing a patent esophagus.

The adult esophagogastroduodenoscopy (EGD) was attempted, which showed a benign-appearing severe stenosis 21 cm from the incisors. There was no pinhole, and the scope could not pass (Figure 1). The scope was changed to the ultraslim scope, which also failed to pass the stenosis, and an attempt to probe a 0.035-inch wire was unsuccessful. Another trial was performed via the PEG tube gastrostomy track where an ultraslim gastroscope was introduced through the stomach to the esophagus. The scope failed again to pass through complete stenosis, which seemed similar to an esophageal septum/thin membrane. Therefore, the CARD technique was performed the next day by 2 endoscopists. An ultraslim gastroscope was introduced through the PEG tube track and advanced to the level of obstruction in the proximal esophagus. Simultaneously, the adult EGD was introduced through the mouth until it reached the level of the esophageal obstruction. The transillumination from the adult EGD was seen by the ultraslim scope on the opposite side (Figure 2). Using this transillumination as a guide, a needle knife was introduced down to the level of the stricture. Free needle septotomy was performed under the guidance of both the transillumination and under direct visualization (Figure 3). The septotomy was preformed successfully without bleeding. A 0.035-inch Jagwire was advanced through the needle knife to the distal side of the esophagus. A Savary wire was advanced under the direct vision of the adult gastroscope through the septotomy, and the adult gastroscope was removed (Figure 4). Dilation was performed with a Savary dilator with no resistance at 5, 7, and 9 mm under fluoroscopy and endoscopy guidance with the ultraslim scope situated in the distal esophagus (Figure 5). The procedure went without complications and was well-tolerated by the patient. EGD was repeated 2 weeks later and showed partial stricture with normal mucosa, which was traversed with the ultraslim gastroscope, and a repeat dilation using a through-the-scope 9 mm balloon was performed (Figure 6).

## DISCUSSION

Complete esophageal stenosis after radiation therapy is not uncommon and is considered a serious adverse event that results in significant morbidity. Before the endoscopic era, management was mainly surgical and sometimes required esophagectomy, which carries high rates of mortality.^6^ The management of complete esophageal stenosis differs from partial stenosis where the antegrade dilation approach is considered the mainstay treatment. The straight antegrade dilation cannot be used with complete luminal occlusion and inability to traverse the stenosis. To overcome this issue, many case series started to use the CARD technique as a treatment for complete esophageal stenosis, providing an alternative to surgical interventions. Those case series showed the CARD procedure was associated with a high success rate, low risk of complications, and improved quality of life. Complications described in those series were mainly in the form of dehiscence of the gastrostomy tract, pneumomediastinum, and perforation.^5^

After antegrade dilation failed in our patient, we attempted the CARD technique. The introduction of the gastroscopes at both ends of the stenosis provided a good visibility during the whole procedure. We used the adult size gastroscope for the antegrade approach and the ultraslim gastroscope for the retrograde approach. This minimized the risks of gastrostomy tract dehiscence and allowed for an easier access to the esophagus from below the stenosis. In the previous cases where the CARD technique was used to treat complete esophageal obstruction, a glidewire was used most of the time to pull the stenotic tissue proximally, making a bulging tissue which is then grasped and removed with a biopsy forceps.^7^ Other techniques that were described in the past included counterpressure forceps, blunt dissection, and direct puncture.^8^ However, we used the needle knife instead to do the septotomy under direct visualization and transillumination, increasing the success rate and reducing the risk of complications. To our knowledge, this is a very rare case where the CARD technique was used with the use of needle knife septotomy to treat complete benign esophageal stenosis. This technique is probably safe, effective, and minimally invasive and provides hope to be used as an alternative for traditional surgical techniques.

## DISCLOSURES

Author contributions: Conception and design: W. Sayeh. Drafting of the article: W. Sayeh, J. Burlen, and S. Ghazaleh. Critical revision of the article for important intellectual content: All authors. Study supervision: A. Tsang, M. Heif, and T. Alhmoud. Final approval of the article: All authors. W. Sayeh is the article guarantor.

Financial disclosure: None to report.

Informed consent was obtained for this case report.
